# Effects of foam rolling and the knowledge-to-action gap: are practitioners’ beliefs supported by the evidence? An international survey study

**DOI:** 10.1186/s13102-025-01514-7

**Published:** 2026-01-13

**Authors:** Stanislav Dimitri Siegel, José Afonso, Ewan Thomas, Mareike Sproll, Astrid Zech, Gerit Plöschberger, Andreas Konrad, David G. Behm, Jan Wilke, Robert Schleip, Konstantin Warneke

**Affiliations:** 1 Institute of Human Movement Science and Exercise Physiology, Friedrich Schiller University Jena, Seidelstraße 20, Jena, 07749 Germany; 2 Institute of Human Movement Science, Sport and Health, University of Graz, Graz, Austria; 3 Centre for Research, Education, Innovation and Intervention in Sport (CIFI2D), Faculty of Sport, University of Porto, Rua Dr. Plácido Costa, 91, Porto, 4200-450 Portugal; 4 Department of Psychology, Sport and Exercise Sciences Research Unit, Educational Science and Human Movement, University of Palermo, Palermo, Italy; 5 School of Human Kinetics and Recreation, Memorial University of Newfoundland, St. John’s, Newfoundland and Labrador Canada; 6 Department of Human Movement Science and Exercise Physiology, University of Hamburg, 20148 Hamburg, Germany; 7 Department of Neuromotorics and Movement, University of Bayreuth, Bayreuth, 95447 Germany; 8 Department of Sport and Health Sciences, Technical University of Munich, Munich, 80333 Germany

**Keywords:** Foam rolling, Survey, Education, Massage, Roller massager, Self-myofascial release exercises

## Abstract

Foam rolling (FR) is commonly used in health and sports settings, yet it remains unclear how well current practice aligns with scientific evidence. We synthesised the available research on FR and compared it with practitioners’ knowledge across professions and language-based cultural spheres.

The evidence consistently supports acute increases in range of motion (ROM), short-term pain reduction, and transient improvements in muscle stiffness and blood flow. However, evidence for performance enhancement, injury prevention, and “fascial adhesion release” is limited or inconclusive, and data on safety are scarce.

A cross-sectional online survey (*n* = 452; conducted in German, Italian, Portuguese, Spanish, and English-speaking countries) showed that only 2 of 15 evidence-based items reached the 80% correct response threshold. A simple majority answered just 10 items in accordance with current evidence, indicating substantial knowledge gaps. Accuracy varied by profession and cultural sphere.

Overall, while FR is effective for acute ROM gains and pain relief, current beliefs about performance and long-term effects are not supported by robust evidence. The mismatch between research and practice highlights the need for clearer communication of findings, accessible continuing education, and evidence-based guidelines.

Identified research gaps do not allow an appropriate judgement of the responses. This research gap calls for future research, while substantial effort should be invested into science communication to reach a broader audience.

**Supplementary Information:**

The online version contains supplementary material available at 10.1186/s13102-025-01514-7.

## Introduction

In empirical and applied science such as health science, one crucial, if not the most important task is to generate new insights through experimental research and to translate these findings into practice effectively [1, 2]. This process, known as knowledge transfer, is crucial in health-related disciplines such as medicine, physiotherapy, and sports science [3, 4]. However, it is often slow and ineffective [5]. In health research, it takes an estimated average of 17 years for results to reach practitioners or patients [6]. Therefore, identifying knowledge gaps among practitioners may be the first step to improve the knowledge transfer from research to practice.

One such topic in health related sciences, including physiotherapy and sports science, is foam rolling (FR). It can be described as tool-assisted self-massage of the soft tissue that is commonly applied with various objectives. In detail, out of 685 respondents, about 70% used FR in their exercise routines to accelerate recovery (72.4%), reduce pain (68.6%) and increase ROM (64.9%) as the most frequent responses [7], drawing a positive picture of FR effects, which is, in parts supported by evidence. In warm-up routines, up to date evidence indicates FR acutely enhanced range of motion (ROM) [8, 9] with accompanying stiffness reductions [10], increased passive peak torque (PPT) [11]. These effects were comparable to those observed after stretching [12], while not being accompanied by performance impairements [13, 14] a considerable assumed disadvantage of extensive stretching [15, 16]. Additionally, research proposed benefits in recovery and cool-down routines [17] and in long-term programs aimed at improving ROM [9]. Proposed FR benefits extend beyond ROM. Some studies have suggested that it may improve cardiovascular health by reducing arterial stiffness and blood pressure, positioning it as a “promising alternative to aerobic exercise” [18]. However, it is also widely used based on the assumption that applying targeted pressure to muscles and surrounding connective tissues helps treating musculoskeletal pain and myofascial trigger points [19].

Despite its widespread applications in performance enhancement, rehabilitation, and prevention, critical questions remain: do common beliefs align with the current evidence? Warneke et al. [20] demonstrated that movement experts, including physiotherapists and sports scientists, often hold beliefs about stretching that do not align with current research. Their survey revealed that for only 10 out of 22 evaluated effects, practitioners’ assumptions matched the scientific evidence, highlighting a significant gap between empirical findings and applied practice. This example illustrates that even well-established interventions can be subject to misconceptions, underscoring the need for improved science communication and implementation in the field. Given the widespread use of FR and its growing role in both, clinical and performance settings, it is important to investigate whether such evidence-practice gaps exist for FR. Therefore, this study aimed to assess and compare evidence awareness among exercise and health professionals regarding foam rolling.

## Methods

### Ethics and design

To comprehensively evaluate FR practices from both scientific and applied perspectives, this study followed a two-step approach. Firstly, an evidence synthesis of the scientific literature was performed which was secondly followed by a structured practitioner survey.

In the first phase, existing scientific evidence on FR applications - such as improving range of motion, enhancing performance, supporting recovery, and promoting overall health - was systematically analyzed by reviewing relevant systematic reviews with and without meta-analyses. This step was performed to provide a solid baseline for categorization of the collected responses in the survey. This design was used in similar, up-to-date surveys before [20, 21]. If a meta-analysis or systematic review was not identified, a search was conducted to identify relevant clinical and randomized controlled trials (RCT).

In the second phase, a structured online survey was conducted to collect information about practitioners’ evidence beliefs. The original survey was drafted in English and subsequently translated into Spanish, Italian, and German by native speakers. These translations ensured broad accessibility and facilitated recruitment of a diverse audience.

Data collection started in September 2024 and ended in March 2025. The study received ethical approval from the local ethics committee (GZ. 39/227/63 ex 2023/24) and all participants provided digital informed consent prior to participation. To ensure data privacy, all responses were collected anonymously and handled in accordance with applicable data protection regulations. Participants were informed that their data would be used solely for research purposes and that they could withdraw from the study at any time without providing a reason.

### Evidence synthesis (study part 1)

In the first part, three investigators (SS, GP, MS) independently conducted a comprehensive literature search between August and September 2024 to identify systematic reviews (with or without meta-analyses) addressing the acute and/or chronic effects of FR on several performance- and health-related parameters. For instance, to explore the acute impact of FR on flexibility, the following search terms were utilized in PubMed: Foam rolling[Title/Abstract] AND (flexibility[Title/Abstract] OR ROM[Title/Abstract] OR “range of motion”[Title/Abstract]) AND (“systematic review”[Title/Abstract] OR “meta-analysis”[Title/Abstract]). Similar searches were conducted for both acute and chronic effects by incorporating relevant keywords such as (acute OR immediate OR short-term) and (chronic OR long-term). A manual search was additionally conducted via Google Scholar (first 500 hits), and the reference lists of all included studies were checked for further relevant publications. Inclusion criteria comprised systematic reviews (with or without meta-analysis) assessing FR interventions for the respective parameter. If no systematic reviews were available, RCTs were included. To reduce the influence of isolated findings and allow for basic synthesis, a minimum of two RCTs addressing the same outcome parameter was required. This threshold does not reflect evidence quality per se but was applied to enhance the interpretability and consistency of the results. Narrative reviews were not considered as primary sources of evidence but were consulted to provide contextual information or identify research gaps when no high-quality evidence was available. Eligible publications had to be available in English or German. The systematic search included the following topics: (1) physiological and performance-related effects, such as flexibility, tissue stiffness, warm-up effects, risk of injury, and fascial adhesion; (2) recovery-related aspects, including musculoskeletal pain, blood flow, and general recovery effects; (3) moderating variables, such as the effect of material properties of the foam roller and exercise intensity, and (4) safety considerations, including contraindications for use and practical recommendations. Based on the evidence gathered, the effects of FR were categorized as either negative, no effect, or positive. This classification was derived from the results of the included meta-analyses (statistical significance of the summary effect sizes) or the overarching conclusions drawn in systematic reviews. If multiple meta-analyses reported consistent positive effects, the overall evidence was classified as positive. If meta-analyses yielded conflicting outcomes (e.g., similar numbers of positive and negative findings), the effect was considered inconclusive (no effect). Predominantly negative outcomes across multiple studies resulted in a classification as negative. When insufficient evidence (fewer than two RCTs or no systematic review) was available for a specific research question, this topic was excluded from classification and statistical analysis. It should be noted that “no effect” in this context does not imply a complete absence of effect but rather refers to statistically non-significant or trivial findings.

### Survey (study part 2)

#### Participants

The questionnaire was made available online via Google Forms (Google LLC, Mountain View, CA, USA) and distributed using a multi-channel recruitment approach. Each author shared the survey within their respective academic institution, and additional universities and educational institutions offering programs in sports science, physiotherapy, and related fields. Institutions were selected based on three predefined criteria: (1) the presence of relevant academic programs, (2) publicly accessible contact channels suitable for survey dissemination (e.g., departmental mailing lists, program coordinators, student offices), and (3) existing informal academic contacts within the authors’ professional networks. Universities that did not meet these criteria, lacked accessible dissemination pathways, or did not respond to initial contact attempts were not included. Although online recruitment strategies inherently carry a risk of selection bias and self-selection effects, combining academic and practice-oriented channels helped maximize the reach and representativeness of the sample. Similar multi-channel approaches have been successfully utilized in previous research [22, 23]. The study surveyed 452 adults from different countries over six months, from September 14, 2024 to March 14, 2025. Participants provided informed consent digitally. Most respondents were aged 18–30 (*n* = 327), with 76 aged 30–40, 30 aged 40–50, 12 aged 50–60, and 7 over 60 years. The majority resided in German-speaking countries (*n* = 272, Germany/Austria) or Italian-speaking countries (*n* = 117, Italy), with smaller groups from English-speaking countries (*n* = 38, Canada/United States) and Portuguese- or Spanish-speaking countries (*n* = 25, Portugal/Spain). Professional backgrounds included individuals still in training with no specific professional experience (*n* = 148), multiple relevant professional qualifications (*n* = 130), trainers (*n* = 76), sports scientists (*n* = 63), therapists (*n* = 28), and other professional backgrounds (*n* = 7).

#### Questionnaire

In contrast to Bartsch et al. [7], the online questionnaire was not designed to evaluate the goals of practitioners when using FR. The survey was conducted to explore health professionals’ beliefs of the evidence on FR and was developed through a consensus process among the authors stemming from the literature findings from the first phase of the study (see supplemental material). To ensure clarity and comprehensibility, the questionnaire underwent face validation by being sent to three representatives of the target population (physiotherapists, fitness coaches, and sports scientists) with at least 10 years of practical experience. These individuals independently reviewed the items and provided feedback, which was incorporated to refine and improve the final version of the instrument.

The finalized questionnaire primarily consisted of two tables corresponding to different FR applications (acute and chronic). Each table listed identified areas of application with three response options (positive, negative or no effect) for each category. In the questionnaire, these categories were briefly explained for each item (e.g., “positive” = reduction in stiffness), to ensure consistent interpretation by participants. The topics covered were flexibility, tissue stiffness, warm-up effects, injury risk, fascial adhesion, recovery, musculoskeletal pain, blood flow, contraindications for the application, the effect of material properties of the foam roller, intensity, and practical recommendations. Additionally, the survey included questions on respondents’ demographics, educational background, and professional experience, such as their age, highest educational qualification, and years of work experience in specific fields. Participants were also asked about the target groups they work with, the educational channels they use to stay informed about scientific knowledge, and whether they regularly engage in FR or self-massage.

#### Statistics

Descriptive statistics, including absolute and relative frequencies, were used to analyze the survey data. To evaluate whether participants responses aligned with existing scientific evidence on FR, a chi-square (χ²) test for proportions (goodness-of-fit test) was employed. This test assumes that the expected frequencies in each category are sufficiently large (commonly at least 5) to ensure validity. If this assumption was violated, Fishers exact test for multi-category distributions was applied. These methods ensured statistical validity even in cases involving small sample sizes or rare events.

When analyzing responses classified as either “in accordance with the evidence” or “not in accordance with the evidence,” an 80/20 distribution was assumed as the baseline, as participants, primarily health and sports professionals, were expected to demonstrate a high level of knowledge aligned with the current evidence. To facilitate comparability with previous studies [20, 21], a second threshold reflecting 50/50 awareness was additionally calculated. This threshold was examined descriptively and not subjected to statistical testing. The χ² test was used to determine whether the observed proportion of correct responses (e.g., identifying FR as beneficial) significantly deviated from this expected ratio. This allowed us to assess whether a majority of participants demonstrated beliefs consistent with the scientific evidence.

To compare response patterns between professional groups and cultural spheres, the χ² test of independence was also applied. Due to the low frequency of some categories, professional groups were consolidated into three broader categories-Other, Scientific Background, and Practical Background- to enable a more valid statistical analysis. Participants with a degree in sports science but no specific occupational information were assigned to the Scientific Background category. Those working in practical roles, such as trainers or instructors, were grouped under Practical Background, while the “Other” category included individuals with no relevant experience or those working in unrelated fields. We also clustered countries by language (e.g., German-speaking countries), used here as a practical proxy for shared cultural and educational backgrounds, while mitigating potential biases introduced by questionnaire translation. This strategy was used to reach more reasonable sample sizes per group, as clustering per country would result in even more heterogeneity.

The original data set and distribution of responses can be found in the supplemental material. A p-value of < 0.05 was considered statistically significant. All analyses were performed using R (Version 4.4.3; R Core Team, 2025) within RStudio (Version 2024.12.1 + 563; Posit, 2025).

## Results of the literature search

### Evidence synthesis

#### Range of motion and performance

A total of 19 systematic reviews (11 of which included meta-analyses) addressed FR-related topics for which the results are presented in Table 1. Eight reviews examined the acute effects of FR on flexibility (four meta-analyses: [8, 12, 32, 33]; four systematic reviews: [17, 25, 34, 37]), and four evaluated its long-term impact on ROM (two meta-analyses:[9, 12]; two systematic reviews: [25, 36]). Across these reviews, evidence consistently suggests that FR acutely increases ROM. Seven reviews investigated acute performance outcomes such as strength and power (three meta-analyses: [10, 32, 35]; four systematic reviews: [34, 36–38]), while one meta-analysis addressed long-term performance [9]. Due to partially conflicting findings across reviews, the overall evidence was classified as showing no clear effect.

#### Stiffness

One meta-analysis focused on acute muscle stiffness without a significant effect [10]. However, a recently conducted meta-analysis by Wilke et al. [32], which included 18 studies, indicates a significant reduction in muscle stiffness as a result of FR. Long-term muscle stiffness was investigated in two RCTs, both showing no significant effects [11, 33].

#### Recovery and pain

Mixed but generally positive recovery benefits were reported by five reviews (four meta-analyses: [32, 35, 50, 52]; one systematic review: [17]). Eight reviews consistently reported short-term reductions in musculoskeletal pain (two meta-analyses: [35, 52]; six systematic reviews: [17, 34, 37, 38, 51, 53]). For long-term pain reduction, two reviews (one meta-analysis: [44]; one systematic review: [51]) concluded that there were no significant effects. However, it should be noted that this analysis also included post-exercise pain (like delayed onset muscle soreness). One recent meta-analysis comparing FR to other warm-up routines found no superiority of FR [25].

#### Fascial adhesions

No systematic reviews in this topic were found. Four RCT [34–37] and one narrative review [38] suggest a positive effect of FR on fascial adhesion. Although standardized definitions and measurement methods for fascial adhesions are still lacking, evidence indicates improved fascial gliding between muscles and overlying fascial layers. A Delphi consensus outlined contraindications for FR use in individuals with open wounds, fractures, or severe health conditions, though no adverse events have been reported [24, 54].

#### Blood flow

No systematic review was found for FR effects on blood flow parameters. Four non-randomized trials [50–53] and six RCTs consistently found acute improvements in local blood flow [44–49].

#### Moderators for effects

No systematic review was conducted on moderator. Two trials explored foam roller material properties [55, 56] and different pressure intensities [57, 58], indicating firmer rollers slightly enhance flexibility without significantly affecting performance outcomes. One RCT explored injury incidence, suggesting potential benefits of FR as part of a broader strength-training program, though no definitive evidence supports its isolated use for injury prevention [59].

#### Evidence based response classification

**Fig. 1:**
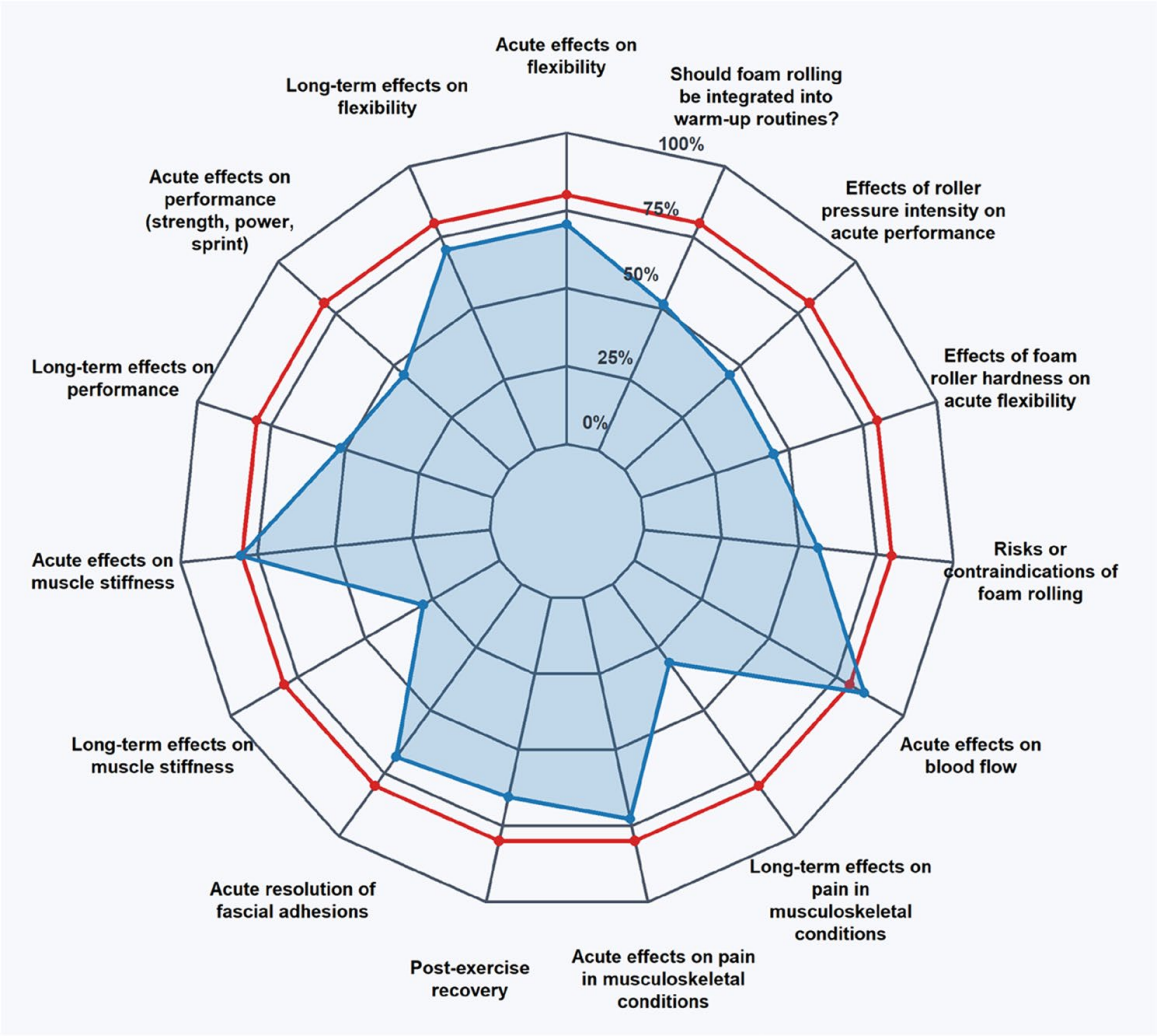
Spider chart showing the proportion of respondents whose answers in all subject areas corresponded with scientific findings (blue area and dots). The red line marks the statistical threshold of 80%. Abbrevation: ROM, range of motion

Based on these 41 articles (19 systematic reviews, 11 meta-analyses, one narrative reviews, one Delphi consensus and 15 RCTs), FR effects were classified as “positive”, “no effect”, or “negative” depending on the outcome measure. Participants’ responses were categorized as “in accordance with the evidence” (correct) or “not in accordance with the evidence” (incorrect). For a transparent classification the currently available evidence is synthesized in Table 1; Fig. 1. For several outcome domains, the number of available studies was insufficient to draw evidence-based conclusions including injury rate, injury prevention, fascial adhesion prevention, and long-term effects of fascial adhesions. Consequently, these were excluded from the final analysis due to a lack of sufficient scientific evidence. While these topics were part of the questionnaire to reflect practitioners’ perspectives, the current evidence base did not meet the predefined criteria for inclusion. Participant responses to these questions can be found in the supplemental material.

**Table 1:** Summary of evidence synthesis and participant responses per research question

Question	*N* (Correct)	Relative *N* (%)	*N* (False)	Relative *N* False (%)	References	Synthesis of evidence
Acute effects on flexibility	319	70.6	133	29.4	**Meta-analysis**: Wilke et al. [8], Skinner et al. [24], Konrad et al. [12], Warneke et al. [25] **Systematic review**: Cheatham et al. [26], Grieve et al. [27], Hendricks et al. [17], Hughes & Ramer [28]	**Positive effect**: Foam rolling increases short-term flexibility compared to no control or passive control, with no difference to other interventions like static stretching.
Long-term effects on flexibility	294	70.6	158	35.0	**Meta-analysis**: Konrad et al. [9], Konrad et al. [12] **Systematic review**: Grieve et al. [27], Pagaduan et al. [29]	**Positive effect**: ≥4 weeks of foam rolling can increase flexibility compared to passive control.
Acute effects on performance (strength, power, sprint)	206	45.6	246	54.4	**Meta-analysis**: Glänzel et al. [10], Skinner et al. [24], Wiewelhove et al. [30] **Systematic review**: Martinez-Aranda et al. [31], Pagaduan et al. [29], Hughes & Ramer [28], Cheatham et al. [26]	**No effect**: Foam rolling does not improve acute performance compared to passive control.
Long-term effects on performance	233	51.5	219	48.5	**Meta-analysis**: Konrad et al. [9]	**No Effect**: No long-term performance improvements from foam rolling compared to passive control.
Acute effects on muscle stiffness	364	80.5	88	19.5	**Meta-analysis**: Glänzel et al. [10], Wilke et al. [32]	**No Effect**: Foam rolling reduce acutely the muscle stiffness.
Long-term effects on muscle stiffness	129	28.5	323	71.5	**RCT**: Kiyono et al. [11], Kasahara et al. [33]	**No Effect**: No evidence for long-term effects on muscle stiffness from foam rolling.
Acute resolution of fascial adhesions	310	68.6	142	31.4	**RCT**: Krause et al. [34], Griefahn et al. [35], Griefahn et al. [36], Nakai et al. [37] **Narrative review**: Behm & Wilke [38]	**Positive Effect**: To date, there is no consensus on a precise definition of fascial adhesions. Most researchers refer to alterations of gliding between the fascial layers. A couple of trials have demonstrated improved gliding following FR intervention.
Post-exercise recovery	296	65.5	156	34.5	**Meta-analysis**: Medeiros et al. [39], Skinner et al. [24], Wiewelhove et al. [30], Zhou et al. [40] **Systematic review**: Hendricks et al. [17]	**Positive Effect**: The majority of studies show recovery measured by performance, pain, muscle swelling benefits compared to passive control, but the results are inconsistent.
Acute effects on pain in musculoskeletal conditions	329	72.8	123	27.2	**Meta-analysis**: Wiewelhove et al. [30], Zhou et al. [40] **Systematic review**: Hughes & Ramer [28], Martinez-Aranda et al. [31], Habscheid et al. [41], Hendricks et al. [17], Santos et al. [42], Cheatham et al. [26]	**Positive Effect**: Foam rolling reduces acute musculoskeletal pain (e.g. muscle soreness) compared to no control or passive control. However, no effects are available for chronic and acute musculoskeletal pain conditions (e.g. low back pain).
Long-term effects on pain in musculoskeletal conditions	140	31.0	312	69.0	**Meta-analysis**: Chen et al. [43] **Systematic review**: Santos et al. [42]	**No Effect**: Foam rolling additionally to standard treatment (e.g. manual therapy, exercise) does not reduce long-term musculoskeletal pain compared to standard or sham treatment over 8 weeks. However, the results are inconsistent.
Acute effects on blood flow	386	85.4	66	14.6	**RCT**: Padron-Cabo et al. [44], Okamoto et al. [45], Alonso-Calvete et al. [46], Schroeder et al. [47], Yoshimura et al. [48], Gordon et al. [49] **Clinical trail**: Shu et al. [50], Hotfiel et al. [51], Schroeter et al. [52], Lai et al. [53]	**Positive Effect**: The majority of studies with pre-post design indicate that foam rolling induces acute blood flow improvement.
Risks or contraindications of foam rolling	254	56.2	198	43.8	**Meta-analysis**: Skinner et al. [24] **Delphi consensus**: Bartsch et al. [54]	**Yes**: Avoid foam rolling in cases of open wounds, fractures, or severe conditions. However, there are no studies that reported adverse effects of foam rolling.
Effects of foam roller hardness on acute flexibility	203	44.9	249	55.1	**RCT**: Cheatham et al. [55] **Clinical trail**: Curran et al. [56]	**Positive Effect**: There are indications that rigid foam rollers improve flexibility more than softer ones, and that surface characteristics such as grooves or spikes may further influence the effects by modifying pressure distribution and tissue stimulation.
Effects of roller pressure intensity on acute performance	206	45.6	246	54.4	**RCT**: Grabow et al. [57], Hirose et al. [58]	**No Effect**: Current evidence from two studies suggests that different foam rolling intensities do not significantly affect range of motion or performance outcomes.
Should foam rolling be integrated into warm-up routines?	233	51.5	219	48.5	**Meta-analysis**: Warneke et al. [25]	**No**: Foam rolling does not appear to have specific advantages over other warm-up methods, such as cycling or dynamic stretching. While it may still be included in a warm-up routine, current evidence does not support a superior effect.

### Survey results

#### Personal background

Participants reported obtaining scientific knowledge predominantly through social media platforms (*n* = 294), scientific databases such as PubMed and Google Scholar (*n* = 291), and general search engines like Google (*n* = 244). Other frequently used educational sources included online courses (*n* = 108), colleagues (*n* = 107), professional training sessions (*n* = 11), and books (*n* = 4). Regarding their own exercise frequency, most respondents trained 3–4 times per week (*n* = 133), followed by 4–5 times (*n* = 100), 2–3 times (*n* = 93), more than 5 times (*n* = 76), and 1–2 times (*n* = 50). Warm-up techniques commonly utilized were mobilization exercises (*n* = 254), dynamic stretching (*n* = 244), jogging (*n* = 161), static stretching (*n* = 98), sport-specific warm-ups (*n* = 82), general unspecific warm-ups (*n* = 80), FR (*n* = 58), and progressive load increases (*n* = 41). For recovery, stretching was the most widely used method (*n* = 303), followed by protein intake (*n* = 199), FR (*n* = 157), massage guns (*n* = 87), movement (*n* = 74), heat therapy (*n* = 63), cryotherapy/ice baths (*n* = 49), breathing exercises (*n* = 45), and compression boots (*n* = 21). Most participants did not regularly use FR or self-massage tools before or after training (*n* = 281). However, 171 participants reported regular use.

#### Effects of foam rolling

According to chi-square test results (*p* < 0.05), only two of 15 evaluated questions surpassed the 80/20 threshold while in eight questions, the majority (> 50%) responded correctly. Specifically, the questions on acute blood flow and stiffness effects achieved the highest alignment, with 85.4% and 80.5% correctly identifying FR’s positive influence, respectively. Although a majority correctly recognized acute (70.6%), long-term (70.6%) flexibility and fascial adhesions (68.6%) effects, these percentages still fell short of the benchmark. In contrast, participant understanding of performance-related outcomes was limited. Only 45.6% identified no acute effects and 51.5% correctly and recognized no long-term performance improvements which only slightly surpassed the lower 50% benchmark. Only 28.5% acknowledging no long-term effects on muscle stiffness. Opinions regarding the inclusion of FR in warm-up routines were evenly divided, with 51.5% supporting inclusion despite no superiority over alternative methods. Awareness of post-exercise recovery (65.5%) and acute musculoskeletal pain reduction (72.8%) approached but did not meet the benchmark. However, only 31.0% recognized no long-term benefits for pain reduction. Additionally, just 44.9% correctly acknowledged foam roller hardness positively influencing acute flexibility, and 45.6% correctly identified that roller pressure intensity does not significantly affect acute performance. Finally, 56.2% were aware of potential risks or contraindications associated with FR.

#### Differences between professions

**Fig. 2:**
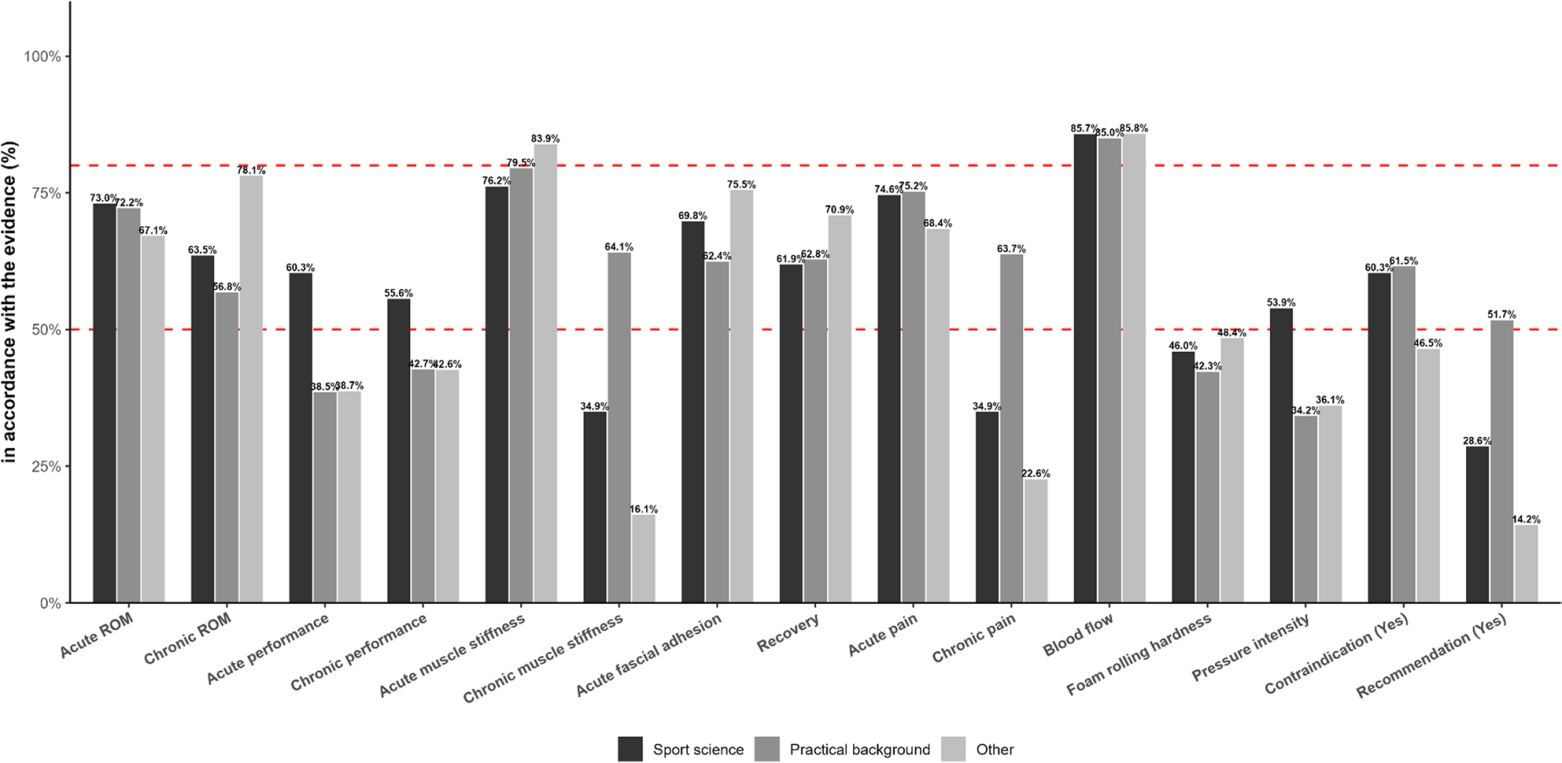
Proportion of respondents whose answers were in accordance with the sciecsports science, practical background, other). Bars show percentages; values are printed above each bar. Dashed red reference lines mark 50% and 80%. Abbreviation: ROM, range of motion

A comparison of the professional groups, namely sports scientists, practitioners, and individuals in training without a practical or clinical sports or health science background - revealed differences in the proportion of correct responses to questions on FR (Tables 2 and 3; Fig. 2)). There were no significant variations between the three groups regarding the acute questions on flexibility (*p* = 0.499), muscle stiffness (*p* = 0.242), blood flow (*p* = 0.976), musculoskeletal pain (*p* = 0.314), and recovery (*p* = 0.207). In contrast, the distribution of correct answers for long-term effects differed significantly for flexibility (*p* < 0.001), muscle stiffness (*p* < 0.001), and pain (*p* = 0.020). For long-term flexibility (correct answer “positive”), 63.5% of sports scientists, 56.8% of practitioners, and 78.1% of those in training selected the correct option. For chronic muscle stiffness (“no effect”), correct response rates were 34.9% for sports scientists, 35.0% for practitioners, and 16.1% for the Other group. Furthermore, the acute fascial adhesions item varied significantly (*p* = 0.027), specifically 69.8% of sports scientists, 62.4% of practitioners, and 75.5% of the Other group gave the correct answer. Regarding chronic pain (“no effect”), 34.9% of sports scientists, 35.5% of practitioners, and 22.6% of the Other group answered correctly. The acute question on fascial adhesions differed significantly between groups (*p* = 0.027); specifically, 30.2% of sports scientists, 36.8% of practitioners, and 23.9% of the Other group correctly selected “no effect”. Performance-related questions also showed notable differences in both acute (*p* = 0.014) and long-term contexts (*p* = 0.022). For acute performance (“no effect”), 60.3% of sports scientists, 46.2% of practitioners, and 38.7% of the Other group selected the correct option, whereas long-term performance (“no effect”) was correctly identified by 55.6%, 56.4%, and 42.6%, respectively. Significant variation also emerged in assessing risks or contraindications (*p* = 0.010), for which the correct response is “yes”: 60.3% of sports scientists, 61.5% of practitioners, and 46.5% of the Other group answered correctly. While roller hardness (*p* = 0.489; “positive”) did not differ significantly among groups, there was a significant difference regarding pressure intensity (*p* = 0.012; “no effect”): 53.9% of sports scientists and practitioners selected the correct option, compared to only 36.1% of the Other group. Finally, no statistically significant difference (*p* = 0.101) emerged regarding FR integration into warm-ups (“no effect”), where correctness remained relatively low across all groups.

**Table 2:** Distribution (relative distribution within a profession) of responses by question and profession

Topic	Sports science			Practical background			Other		
	positive effect	no effect	negative effect	positive effect	no effect	negative effect	positive effect	no effect	negative effect
acute ROM ^a^	46 (73.0)	17 (27.0)	0 (0.0)	169 (72.2)	63 (26.9)	2 (0.9)	104 (67.1)	46 (29.7)	5 (3.2)
chronic ROM ^a, b^	40 (63.5)	23 (36.5)	0 (0.0)	133 (56.8)	101 (43.2)	0 (0.0)	121 (78.1)	32 (20.6)	2 (1.3)
acute performance ^a, b^	18 (28.6)	38 (60.3)	7 (11.1)	90 (38.5)	108 (46.2)	36 (15.4)	70 (45.2)	60 (38.7)	25 (16.1)
chronic performance ^a, b^	27 (42.9)	35 (55.6)	1 (1.6)	100 (42.7)	132 (56.4)	2 (0.9)	80 (51.6)	66 (42.6)	9 (5.8)
acute muscle stiffness ^a^	48 (76.2)	7 (11.1)	8 (12.7)	186 (79.5)	44 (18.8)	4 (1.7)	130 (83.9)	22 (14.2)	3 (1.9)
chronic muscle stiffness ^a, b^	40 (63.5)	22 (34.9)	1 (1.6)	150 (64.1)	82 (35.0)	2 (0.9)	126 (81.3)	25 (16.1)	4 (2.6)
acute fascial adhesion ^a, b^	44 (69.8)	19 (30.2)	0 (0.0)	146 (62.4)	86 (36.8)	2 (0.9)	117 (75.5)	37 (23.9)	1 (0.7)
recovery ^a^	39 (61.9)	20 (31.7)	4 (6.3)	147 (62.8)	79 (33.8)	8 (3.4)	110 (70.9)	39 (25.2)	6 (3.9)
acute pain ^a^	47 (74.6)	13 (20.6)	3 (4.8)	176 (75.2)	47 (20.1)	11 (4.7)	106 (68.4)	44 (28.4)	5 (3.2)
chronic pain ^a, b^	39 (61.9)	22 (34.9)	2 (3.2)	149 (63.7)	83 (35.5)	2 (0.9)	117 (75.5)	35 (22.6)	3 (1.9)
blood flow ^a^	54 (85.7)	8 (12.7)	1 (1.6)	199 (85.0)	33 (14.1)	2 (0.9)	133 (85.8)	21 (13.6)	1 (0.7)
foam rolling hardness ^a^	29 (46.0)	30 (47.6)	4 (6.3)	99 (42.3)	113 (48.3)	22 (9.4)	75 (48.4)	53 (34.2)	27 (17.4)
pressure intensity ^a, b^	21 (33.3)	34 (53.9)	8 (12.7)	80 (34.2)	116 (49.6)	38 (16.2)	72 (46.5)	56 (36.1)	27 (17.4)

Abbreviation: *ROM* Range of motion

a χ² goodness of fit test, significant difference (p<0.05) between distributions

b χ² test of independence, significant difference (p<0.05) between professions

**Table 3:** Distribution (relative distribution) of responses by question and profession

Topic	Sports science			Practical background			Other		
	Yes	No	Undecided	Yes	No	Undecided	Yes	No	Undecided
Contraindication ^a, b^	38 (60.3)	25 (39.7)	0 (0.0)	144 (61.5)	90 (38.5)	0 (0.0)	72 (46.5)	83 (53.6)	0 (0.0)
Recommendation ^a^	23 (36.5)	18 (28.6)	22 (34.9)	121 (51.7)	43 (18.4)	70 (29.9)	75 (48.4)	22 (14.2)	58 (37.4)

^a^ χ² goodness of fit test, significant difference (*p* < 0.05) between distributions

^b^ χ² test of independence, significant difference (*p* < 0.05) between professions

#### Differences between language-based cultural spheres

**Fig. 3:**
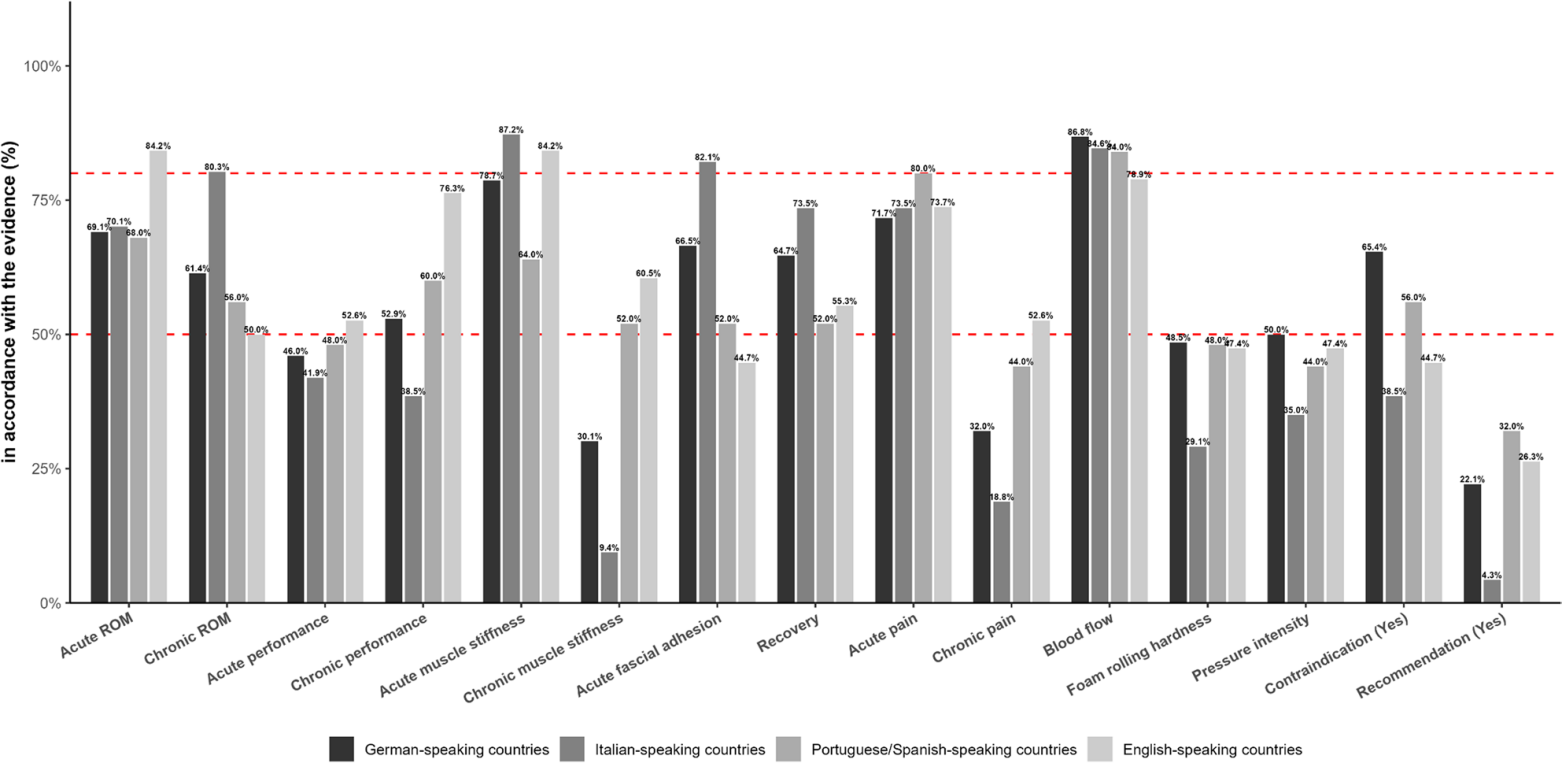
Proportion of respondents whose answers were in accordance with the scientific evidence across all topics (x-axis), stratified by question and language-based cultural sphere (German-speaking countries, Italian-speaking countries, Portuguese-/Spanish-speaking countries, English-speaking countries). Bars show percentages; values are printed above each bar. Dashed red reference lines mark 50% and 80%. Abbreviation: ROM, range of motion

Tables 4 and 5; Fig. 3 illustrate the distribution of responses across language-based cultural spheres for each survey item. While the proportion of participants answering acute flexibility (*p* = 0.287), acute performance (*p* = 0.686), blood flow (*p* = 0.560), and recovery (*p* = 0.067) correctly was largely similar across German-speaking, Italian-speaking, Portuguese-/Spanish-speaking, and English-speaking regions, a significant difference emerged for acute muscle stiffness (*p* = 0.021). Specifically, 78.7% in German-speaking countries, 87.2% in Italian-speaking countries, 64.0% in Portuguese-/Spanish-speaking regions, and 84.2% in English-speaking regions provided the correct “positive effect” response. Marked differences arose regarding acute effects on fascial adhesions (*p* < 0.001), where 66.5% in German-speaking countries, 82.1% in Italian-speaking countries, 52.0% in Portuguese-/Spanish-speaking regions, and 44.7% in English-speaking regions chose the correct “positive effect” answer. By contrast, long-term items revealed more pronounced disparities. Significant differences were found for long-term flexibility (*p* < 0.001), long-term performance (*p* < 0.001), and long-term muscle stiffness (*p* < 0.001). For flexibility, 61.4% of respondents in German-speaking countries, 80.3% in Italian-speaking countries, 56.0% in Portuguese-/Spanish-speaking regions, and 50.0% in English-speaking regions answered correctly (“positive”). Similar variations were evident for long-term performance (“no effect”: 52.9%, 38.5%, 60.0%, and 76.3%) and long-term muscle stiffness (“no effect”: 30.1%, 9.4%, 52.0%, and 60.5%). Differences also emerged regarding long-term pain reduction (*p* < 0.001), with correctness rates ranging from 32.0% in German-speaking countries to 18.8% in Italian-speaking countries, 44.0% in Portuguese-/Spanish-speaking regions, and 52.6% in English-speaking regions. In contrast, acute pain (*p* = 0.833) was answered correctly (“positive”) at similar rates-ranging from 71.7% to 80.0%-across all groups. Further differences were observed concerning risks or contraindications (*p* < 0.001): 65.4% of German speakers, 38.5% of Italians, 56.0% of Portuguese-/Spanish speakers, and 44.7% of English speakers correctly answered “yes.” Meanwhile, rolling intensity (*p* = 0.059) and roller hardness (*p* = 0.102) did not differ significantly between groups. Finally, the warm-up integration question (*p* < 0.001) revealed substantial disparity in “no” responses: only 22.1% of German-speaking countries, 4.3% of Italian-speaking countries, 32.0% of Portuguese-/Spanish-speaking countries, and 26.3% of English-speaking countries selected the correct option.

**Table 4:** Distribution (relative distribution) of responses by question and language-based cultural sphere

Topic	German-speaking countries			Italian-speaking countries			Portuguese-/Spanish-speaking countries			English-peaking countries		
	positive effect	no effect	negative effect	positive effect	no effect	negative effect	positive effect	no effect	negative effect	positive effect	no effect	negative effect
acute ROM ^a^	188 (69.1)	80 (29.4)	4 (1.5)	82 (70.1)	33 (28.2)	2 (1.7)	17 (68.0)	7 (28.0)	1 (4.0)	32 (84.2)	6 (15.8)	0 (0.0)
chronic ROM ^a, b^	167 (61.4)	104 (38.2)	1 (0.4)	94 (80.3)	22 (18.8)	1 (0.9)	14 (56.0)	11 (44.0)	0 (0.0)	19 (50.0)	19 (50.0)	0 (0.0)
acute performance ^a^	93 (34.2)	125 (46.0)	54 (19.8)	62 (53.0)	49 (41.9)	6 (5.1)	10 (40.0)	12 (48.0)	3 (12.0)	13 (34.2)	20 (52.6)	5 (13.2)
chronic performance ^a, b^	122 (44.9)	144 (52.9)	6 (2.2)	67 (57.3)	45 (38.5)	5 (4.3)	9 (36.0)	15 (60.0)	1 (4.0)	9 (23.7)	29 (76.3)	0 (0.0)
acute muscle stiffness ^a^	214 (78.7)	46 (16.9)	12 (4.4)	102 (87.2)	12 (10.3)	3 (2.6)	16 (64.0)	9 (36.0)	0 (0.0)	32 (84.2)	6 (15.8)	0 (0.0)
chronic muscle stiffness ^a, b^	187 (68.8)	82 (30.1)	3 (1.1)	103 (88.0)	11 (9.4)	3 (2.6)	11 (44.0)	13 (52.0)	1 (4.0)	15 (39.5)	23 (60.5)	0 (0.0)
acute fascial adhesion ^a, b^	181 (66.5)	90 (33.1)	1 (0.4)	96 (82.1)	19 (16.2)	2 (1.7)	13 (52.0)	12 (48.0)	0 (0.0)	17 (44.7)	21 (55.3)	0 (0.0)
recovery ^a^	176 (64.7)	85 (31.3)	11 (4.0)	86 (73.5)	26 (22.2)	5 (4.3)	13 (52.0)	12 (48.0)	0 (0.0)	21 (55.3)	15 (39.5)	2 (5.3)
acute pain ^a^	195 (71.7)	66 (24.3)	11 (4.0)	86 (73.5)	25 (21.4)	6 (5.1)	20 (80.0)	4 (16.0)	1 (4.0)	28 (73.7)	9 (23.7)	1 (2.6)
chronic pain ^a, b^	180 (66.2)	87 (32.0)	5 (1.8)	94 (80.3)	22 (18.8)	1 (0.9)	13 (52.0)	11 (44.0)	1 (4.0)	18 (47.4)	20 (52.6)	0 (0.0)
blood flow ^a^	236 (86.8)	35 (12.9)	1 (0.4)	99 (84.6)	17 (14.5)	1 (0.9)	21 (84.0)	2 (8.0)	2 (8.0)	30 (78.9)	8 (21.1)	0 (0.0)
foam rolling hardness ^a, b^	110 (40.4)	132 (48.5)	30 (11.0)	62 (53.0)	34 (29.1)	21 (17.9)	11 (44.0)	12 (48.0)	2 (8.0)	20 (52.6)	18 (47.4)	0 (0.0)
pressure intensity ^a^	81 (29.8)	136 (50.0)	55 (20.2)	66 (56.4)	41 (35.0)	10 (8.6)	10 (40.0)	11 (44.0)	4 (16.0)	16 (42.1)	18 (47.4)	4 (10.5)

Abbreviation: ROM = range of motion

^a^ χ² goodness of fit test, significant difference (*p* < 0.05) between distributions

^b^ χ² test of independence, significant difference (*p* < 0.05) between countries

**Table 5:** Distribution of responses by question and language-based cultural sphere

Topic	German-speaking countries			Italian-speaking countries			Portuguese-/Spanish-speaking countries			English-speaking countries		
	Yes	No	Undecieded	Yes	No	Undecieded	Yes	No	Undecieded	Yes	No	Undecieded
Contraindication ^a, b^	178 (65.4)	94 (34.6)	0 (0.0)	45 (38.5)	72 (61.5)	0 (0.0)	14 (56.0)	11 (44.0)	0 (0.0)	17 (44.7)	21 (55.3)	0 (0.0)
Recommendation ^a, b^	104 (38.2)	60 (22.1)	108 (39.7)	83 (70.9)	5 (4.3)	29 (24.8)	11 (44.0)	8 (32.0)	6 (24.0)	21 (55.3)	10 (26.3)	7 (18.4)

^a^ χ² goodness of fit test, significant difference (*p* < 0.05) between distributions

^b^ χ² test of independence, significant difference (*p* < 0.05) between countries

## Discussion

By assessing awareness of scientific evidence among health professionals and sports scientists, this survey underlines the high demand for improved science communication to optimize performance, prevention, and rehabilitation practice. Our findings are in accordance with the recently performed stretching survey by Warneke et al. [20] in which the authors surveyed 117 movement experts about stretching and outlined unawareness of evidence in the majority of addressed topics. However, the authors used a 50/50 threshold while this survey assumed higher knowledge in movement experts in the topic of FR. To reach comparability, additionally, this threshold was also applied here, weakening the unawareness to the queried topics. Indeed, relevant beliefs are present regarding the effects of FR, emphasizing the need for improved education and dissemination of evidence-based practices across movement-related professions. In addition, the relevance of this topic is underscored by the findings of Cheatham [60], where 629 of 1000 surveyed physiotherapists, fitness professionals, and athletic trainers would prescribe FR at least once per week or even daily for their clients. In contrast to the Bartsch et al. [7] review, the majority of the included sample in the presented study did not use FR regularly in their own training. This discrepancy could explain the worse results in the presented study and it can be hypothesized that applicants inform themselves about the evidence when aiming to include the intervention into their own daily exercise practice, which can enhance commitment.

### Evaluation of the current evidence on foam rolling

Despite the substantial body of evidence on FR, including over 13 meta-analyses published in the past ten years on performance, flexibility, recovery, and pain perception, key questions remain unanswered. There is no evidence on contraindications. Evidence for acute improvements of myofascial adhesions - interpreted here as enhanced fascial gliding - is very limited and of low quality, not least because the underlying concept of “myofascial adhesions” remains poorly defined. Similarly, the effects of FR on the cardiovascular system and its long-term impact on muscle stiffness have not been systematically reviewed, underscoring the need for further research in these areas. While the effects on flexibility and performance are derived from a relatively homogeneous body of research, outcomes related to recovery and pain exhibit greater variability, reflecting a more heterogeneous study landscape. Methodological limitations, including the lack of blinding, placebo controls, and standardized protocols, are prevalent, with PEDro scores ranging from 3 to 7 in the included studies (low - moderate quality of evidence). These factors constrain the interpretability and generalizability of the meta-analytic findings. Moreover, the lack of longitudinal data and multi-arm comparative trials limits the ability to compare FR with other interventions. In clinical practice, time-efficient and effective treatments are essential, raising the question: are there “better” alternatives to FR [25]? Further research should determine whether FR confers unique advantages over conventional methods - such as standard stretching or various warm-up protocols - or merely offers an equivalent alternative. Achieving this will require more robust longitudinal studies focusing on specific markers of fascial structure, muscle stiffness, and recovery. In the absence of such evidence, a critical approach to FR remains advisable, both to avoid misconceptions in professional practice and to prevent unfounded promises of its efficacy.

###  Awareness of foam rolling evidence

Unfortunately, four of 19 questions could not be substantiated with scientific evidence. Eight out of the remaining 15 questions received correct answers from more than 50% of respondents. The results are comparable to a recently conducted survey on stretching, in which sports scientists and physiotherapists also participated; in that study, 10 out of 22 questions were answered correctly by more than 50% of participants [20]. Nevertheless, participants’ responses to these questions were highly heterogeneous, with a noticeable trend toward assuming a positive effect. Topics that are widely covered in the literature and commonly practiced-such as acute and long-term flexibility improvements, post-exercise recovery, and pain perception-showed relatively high accuracy rates (65.5–72.8%). By contrast, questions addressing long-term physiological mechanisms, such as muscle stiffness or pain, revealed broader variability and generally lower correctness rates (28.5–70.6%). This pattern may partly reflect the limited amount of research available on these mechanisms, as the included systematic literature search indicated that fundamental questions - such as how fascia responds to FR and how tissue stiffness may be influenced - remain insufficiently explored. Furthermore, the results of the evidence search suggest that aspects such as potential contraindications have not been adequately explored in the current literature, while being listed as a reason to not implement FR into training routines in previous research [7]. Further practical relevant topics are critically underinvestigated. A prominent example is the claim that FR can “release” fascial adhesions, a notion lacking robust evidence-partly due to the frequent, yet imprecise, use of terms like myofascial release without any demonstrated mechanism of actual tissue release [10, 38]. Survey results of Cheatham and Stull [61] similarly revealed that misconceptions about FR persist among allied health students: about half believed FR could break up scar tissue, while 75% attributed benefits to reduced muscle tension. Such misunderstandings risk leading practitioners to overestimate FR’s structural effects, possibly overlooking more established interventions. These beliefs may also stem from the lack of dedicated FR coverage in professional training programs. Indeed, Cheatham and Stull [61] note that only about half of students in health-related fields in the United States reported exposure to FR during their education. Taken together, these findings highlight the urgent need for more comprehensive and evidence-based education on FR - particularly regarding realistic short-term benefits (e.g., subjective pain relief, transient increases in range of motion) versus the largely unsubstantiated claims of significant long-term structural change.

### Differences in beliefs between professions

Although sports scientists and practitioners generally showed higher overall accuracy than participants without a formal background in sports or health sciences, there was a substantially variation in response accuracy (14–88%) and the difference between groups was smaller than expected. Notably, for 5 out of 15 questions, participants in the “Other” group selected the correct answer more frequently than the experts. One potential explanation lies in the group categorization: the “Other” group also included sports science students without a completed degree, which may have contributed to their relatively strong performance. In addition, the topic of FR may be a contributing factor-its growing popularity and presence in social media, fitness communities, and informal education settings could have made relevant knowledge more broadly accessible. However, Cheatham et al. [62] surveyed practitioners - including physical therapists, athletic trainers, and fitness professionals - regarding their observed effects of FR. About 50% reported increases in ROM and reductions in patient-reported pain, suggesting that practitioners draw on direct field experience when assessing the effectiveness of FR. Overall, these findings highlight the importance of targeted education and knowledge translation for all groups. While university-trained individuals may be more attuned to ongoing research, practitioners’ extensive experience can also yield valuable insights. Future efforts could focus on unifying and updating best practices around FR to ensure that both scientific evidence and practical know-how are effectively shared across professional boundaries.

### Differences in beliefs between cultural spheres

This study marks one of the first attempts to explore FR beliefs across German-, Italian, Portuguese-/Spanish, and English-speaking cultural spheres. Although the majority of participants were from German-speaking (63%) and Italian-speaking countries (28%), with smaller groups representing Portuguese-/Spanish-speaking and the English-speaking countries, these data nonetheless offer initial insights into possible regional differences. When comparing across language groups, participants from the English-speaking countries demonstrated higher accuracy on 5 of the 15 questions, followed by Portuguese-/Spanish-speaking on 4, German-speaking on 4, and Italian-speaking countries on 3. Among the two largest cohorts, German-speaking respondents answered more questions correctly than Italians on 10 out of 15 items. However, these findings should be interpreted with caution, as the distribution of professions was not fully balanced across regions and the sample sizes differed considerably. These factors may have influenced the observed differences and limit the generalizability of the results. Further research with larger and more homogeneous samples is needed to confirm these preliminary observations. Ultimately, a more standardized and evidence-based approach to FR education - both in formal curricula and through continuing professional development - may help address misconceptions and improve the consistency of FR-related knowledge across diverse regions.

### Practical recommendations

The half-life of knowledge refers to the time it takes for existing knowledge to be updated, refined, or replaced by new findings. As Helmrich & Leppelmeier [63] highlight, knowledge does not simply “decay” but evolves through continuous refinement. In practice, this poses a challenge, as professionals must constantly update their knowledge to stay evidence-based. Without effective strategies for knowledge dissemination and education, gaps between research and practice persist, delaying the application of scientific advancements. Regular training and improved science communication are essential to ensure that new insights reach and benefit practitioners effectively. Applying the RE-AIM model (Reach, Effectiveness, Adoption, Implementation, and Maintenance; 5) provides a useful framework for understanding how evidence can be translated into practice. In the context of FR research, the model highlights shortcomings particularly in the dimensions of reach (limited dissemination of evidence), adoption (variable use by practitioners), and implementation (lack of structured educational integration). To address these challenges, more structured continuing education, integration of FR-related evidence into university curricula, and collaboration between researchers and professional associations are needed. Such efforts-while present in some countries-remain fragmented and inconsistent, emphasizing the need for coordinated, cross-national strategies to strengthen evidence-based practice.

### Limitations

This study has several limitations that should be considered when interpreting the findings. Firstly, we do not have precise data on the number of individuals who were initially contacted or received the survey link, which restricts our ability to assess response and selection bias. Additionally, the distribution of professional backgrounds across countries was not systematically controlled, potentially leading to selection bias in country-specific comparisons. Moreover, 60% of participants were from German-speaking countries, while representation from other regions - such as Spain, Portugal, and the United States - was limited. This uneven distribution restricts the generalizability of the results to broader populations. Additionally, while the study included a diverse range of professional groups, the sample sizes for some subgroups were relatively small, potentially affecting the validity of subgroup analyses. Although the questionnaire was not translated into Portuguese, it is worth noting that Portuguese-speaking individuals generally find it easy to understand written Spanish. The survey relied on self-reported data, which may be influenced by biases such as social desirability or inaccurate recall, potentially impacting the accuracy of the responses. Some questions, particularly those addressing physiological mechanisms like fascial adhesions, may have been misinterpreted due to varying definitions and understandings. The beliefs assessment was based on alignment with existing evidence, which is continuously evolving. As such, discrepancies in participants responses may reflect gaps in the dissemination of evidence rather than a lack of understanding. Contextual factors such as the availability of resources, differences in educational curricula, or cultural attitudes toward FR were also not accounted for, which may have influenced participants responses. Lastly, psychological factors such as expectations, placebo effects, and self-fulfilling prophecies may have influenced participants’ responses. A potential survival bias should also be considered, as individuals with particularly strong opinions or positive personal experiences with foam rolling may have been more likely to participate in the survey. Furthermore, the interpretation of some survey items may have varied among respondents. As the questions did not specify a comparator (e.g., foam rolling versus alternative interventions or versus no intervention), participants may have applied different reference frames when assessing the effectiveness of FR. Consequently, the accuracy scores might partly reflect differences in interpretation rather than actual knowledge or belief discrepancies. Although the questionnaire was pre-tested with representatives of the target population (physiotherapists, fitness coaches, and sports scientists) to minimize ambiguity, this limitation is inherent to survey-based research. Finally, the classification of evidence as positive, no effect, or negative was based on the statistical significance reported in the included meta-analyses and systematic reviews. This approach does not account for the magnitude or practical relevance of the reported effects. Therefore, “no effect” in this context should be interpreted as statistically non-significant or trivial findings rather than a complete absence of effect. Future research could benefit from a more nuanced classification system that considers both effect sizes and the quality of the underlying evidence.

## Conclusion

This survey provides the first multi-country comparison of FR beliefs, highlighting variations across professions and regions. Only 9 of the questions were correctly answered by most respondents, with significant gaps in understanding physiological mechanisms like fascial adhesions. While sports science graduates showed better alignment with current evidence, inconsistencies in accuracy persisted. Regional trends offer initial insights but require further validation. These findings emphasize the need for improved education and rigorous research to bridge knowledge gaps and support evidence-based FR practices.

## Supplementary Information

Supplementary Material 1.

Supplementary Material 2.

## Data Availability

The raw datasets generated during and/or analyzed during the current study are not publicly available.

## Abbreviations

ADM: Availability of Data and Material
FR: Foam rolling
GZ: ethics-committee protocol identifier
PEDro: Physiotherapy Evidence Database
PPT: Passive peak torque
R: R statistical-computing environment
RCT: Randomized controlled trial
RE-AIM: Reach, Effectiveness, Adoption, Implementation and Maintenance model ​
ROM: Range of motion
χ²: chi-square statistic (goodness-of-fit / independence test)
