# Supplementary material for: Effects of foam rolling and the knowledge-to-action gap: are practitioners’ beliefs supported by the evidence? An international survey study
Source: BMC Sports Sci Med Rehabil. 2026 Jan 13;18:104. doi: 10.1186/s13102-025-01514-7 (PMC12930799; doi:10.1186/s13102-025-01514-7)
Supplement: Supplementary file 2 — Supplementary Material 2. [file 13102_2025_1514_MOESM2_ESM.docx]

**Supplementary Materials**

**Survey**

Foam rolling: Are practical applications evidence-based?

Declaration of consent and personal data

This consent form is intended to give you an insight into this study and to inform you about what to expect. If you have any questions or would like to know more about this study, please do not hesitate to contact us. Please take enough time to read this text carefully.
 
**Aim of the study:**With this survey study, we want to shed light on the scientific transfer in sports science on the subject of foam rolling. For this purpose, we ask you to provide your opinion on the topic of foam rolling in various settings such as warm-up, self-massage and recovery. The questionnaire will be 100% anonymous and no identification data will be collected.
 
**Procedure of the study:**Participation is possible regardless of location, as the questionnaire is available online. Please read the privacy policy and the consent to participate carefully. If you do not agree to this, please click “no”. This ends your participation. If you wish to participate, please tick the “yes”-button. You will then be forwarded to the questionnaire. First, you will be asked to provide various information about your background (e.g. age, educational qualification, working experience..). 
Afterwards, you will be asked to respond to 18 questions about foam rolling using multiple-choice options. Each question will have three possible answers (positive, no effect, negative). Please select the answer you believe is correct for each question. Participating will take you about 5-10 minutes.

**Inconveniences and risks:**There are no risks or inconveniences, nor can it be traced afterwards who entered which answers. This means that you do not need to worry about your answers being evaluated on a personal basis.
 
**Data protection:**The results cannot be linked to any individual. The overall analysis and results are strictly confidential. No access will be granted to individuals outside the research team. Once you consent to participate, it will no longer be possible to trace or modify your responses, meaning that revision or deletion is not possible.
 
**Voluntary Participation:**All test participants take part voluntarily and have the right to withdraw their participation at any time without giving reasons while filling the answers. In this case, you can also request the deletion of your personal and collected data. After you submit your responses, it is not possible to delete your responses as we have not collected any data on the person.
 
Your signature, in this case “yes”, confirms that you have understood the contents of the declaration of consent.

The study protocol was confirmed by the ethical review board of the University Graz.

1. I read and understood the previous paragraphs on the study procedure, data protection and voluntary participation and want to participate in this study.

Yes

No

1. In which country do you currently live?
2. What is your age?
3. Please provide information on your educational background.

High school diploma/graduation certificate

Bachelor
Master
PhD

1. Do you have working experience in one or more of the following areas:

Sport science

Trainer/Coach

Physiotherapy

Osteopathy

Ergotherapy/Occupational therapy

Sports therapy

Teacher training in physical education and sports

No experience

1. How long was your working experience in this field (in years)?
2. Please provide some information about your (primary) clients.

Professional athletes

Recreational athletes

Patients

Students

1. Which sources do you use to stay informed about the latest scientific findings?

Social Media (Youtube, Instagram, TikTok, etc.)

Scientific Databases (PubMed, Web of Science, Scopus, Google Scholar, etc.)

Search Engines (Google, Bing, Yahoo!, etc.)

Online Courses

Peers (other therapists, coaches, etc.)

None at all

1. Please provide some information on your training frequency (per week).
2. What is your preferred warm-up protocol?

static streching

foam rolling

dynamic stretching

jogging

mobilization

sport-specific

elastic bands

combination of several of the above

1. Do you regularly perform foam rolling or other forms of self-massage (using tools like massage balls) before or after training?

Yes, before training

Yes, after training

Yes, before and after training

No

1. Which methods do you use to support your recovery after training?

stretching

foam rolling

heat therapy

cryotherapy/ice bathing

protein intake

massage gun

compression boots

low-intensity cycling

low-intensity jogging

breathing work

What do you know about foam rolling?

Thank you for participating in our survey. The aim of this questionnaire is to capture your personal assessment and attitude towards the topic of foam rolling, also known as Black Roll. We want to understand how the athletic population perceives this topic and to what extent scientific findings are integrated into training practice.

Please note that this questionnaire is **completely anonymous**. No personal data will be collected that would allow for the identification of your person. For this reason, it will not be possible to correct or delete your answers afterwards. We greatly appreciate your honest and open responses.

1. How does foam rolling **acutely** (immediately afterward) affect joint range of motion?

positively

no effect

negatively

1. How does regular foam rolling affect joint range of motion in the **long term** (chronic effects, after an intervention period of at least 2 weeks)

positively

no effect

negatively

1. How does foam rolling as a warm-up protocol **acutely** (immediately afterward) affect performance (e.g., maximum strength, explosive strength, jumping, and sprinting)?

positively

no effect

negatively

1. How does regular foam rolling affect performance in the **long term** (e.g., maximum strength, explosive strength, jumping, and sprinting)?

positively

no effect

negatively

1. How does foam rolling **acutely** (immediately afterward) affect muscle stiffness?

reduces muscle stiffness

no effect

increases muscle stiffness

1. How does regular foam rolling affect muscle stiffness in the **long term**?

reduces muscle stiffness

no effect

increases muscle stiffness

1. How does foam rolling **acutely** (immediately afterward) affect the release of fascial adhesions ("myofascial release")?

positively

no effect

negatively

1. How does regular foam rolling influence the **long term** reduction of fascial adhesions ("myofascial release")?

positively

no effect

negatively

1. How does regular foam rolling affect the prevention of myofascial adhesions (fascia adhesions)?

positively

no effect

negatively

1. How does foam rolling **acutely** (immediately afterward) after training impact recovery?

positively

no effect

negatively

1. How does foam rolling as a warm-up protocol **acutely** (immediately afterward) affect injury risk?

positively (i.e., reduces injury risk)

no effect

negatively (i.e., increases injury risk)

1. How does regular foam rolling as a warm-up protocol in the **long term** affect injury risk?

positively (i.e., reduces injury risk)

no effect

negatively (i.e., increases injury risk)

1. How does foam rolling **acutely**(immediately afterward) affect pain perception in musculoskeletal conditions (including back pain)?

positively (i.e., reduces perceived pain)

no effect

negatively (i.e., increases perceived pain)

1. How does regular foam rolling in the **long term** affect pain perception in musculoskeletal conditions (including back pain)?

positively (i.e., reduces perceived pain)

no effect

negatively (i.e., increases perceived pain)

1. How does foam rolling **acutely** (immediately afterward) affect blood circulation in the treated body region?

positively

no effect

negatively

1. Are there any contraindications or risks associated with foam rolling (can the use of foam rollers be harmful)?

positively

no effect

negatively

1. How does an increase in foam roller hardness affect **acutely** (immediately afterward) affect joint range of motion?

positively

no effect

negatively

1. How does an increase in pressure intensity on the foam roller affect **acutely** (immediately afterward) strength and performance?

positively

no effect

negatively

1. Would you recommend including foam rolling as part of a warm-up protocol before training?

Yes

No

Unclear/I don’t know

Table 1S Distribution (relative distribution within a profession) of responses by question and profession

| Country | Topic | Therapy  setting | | | Sport  science | | | Coach/  Trainer | | | Multiple | | | No practical  experience | | | Other | | |  |
| --- | --- | --- | --- | --- | --- | --- | --- | --- | --- | --- | --- | --- | --- | --- | --- | --- | --- | --- | --- | --- |
|  |  |  |  |  |  |  |  |  |  |  |  |  |  |  |  |  |  |  |  |  |
|  |  | + | o | - | + | o | - | + | o | - | + | o | - | + | o | - | + | o | - |  |
| German-speaking countries | acute ROM | 14 | 6 | 0 | 30 | 12 | 0 | 19 | 13 | 1 | 58 | 21 | 0 | 64 | 26 | 3 | 3 | 2 | 0 |  |
|  | chronic ROM | 9 | 11 | 0 | 26 | 16 | 0 | 24 | 9 | 0 | 37 | 42 | 0 | 68 | 24 | 1 | 3 | 2 | 0 |  |
|  | acute performance | 8 | 6 | 6 | 8 | 28 | 6 | 18 | 12 | 3 | 19 | 41 | 19 | 40 | 33 | 20 | 0 | 5 | 0 |  |
|  | chronic performance | 7 | 13 | 0 | 17 | 24 | 1 | 21 | 12 | 0 | 28 | 49 | 2 | 47 | 43 | 3 | 2 | 3 | 0 |  |
|  | acute muscle stiffness | 15 | 5 | 0 | 31 | 4 | 7 | 25 | 6 | 2 | 59 | 18 | 2 | 80 | 12 | 1 | 4 | 1 | 0 |  |
|  | chronic muscle stiffness | 13 | 7 | 0 | 28 | 13 | 1 | 26 | 6 | 1 | 45 | 33 | 1 | 73 | 20 | 0 | 2 | 3 | 0 |  |
|  | fascial adhesion prevention | 14 | 6 | 0 | 26 | 15 | 1 | 29 | 4 | 0 | 49 | 29 | 1 | 76 | 17 | 0 | 2 | 3 | 0 |  |
|  | acute fascial adhesion | 13 | 7 | 0 | 30 | 12 | 0 | 22 | 11 | 0 | 46 | 32 | 1 | 67 | 26 | 0 | 3 | 2 | 0 |  |
|  | chronic fascial adhesion | 14 | 6 | 0 | 25 | 17 | 0 | 23 | 9 | 1 | 43 | 36 | 0 | 78 | 14 | 1 | 3 | 2 | 0 |  |
|  | recovery | 12 | 5 | 3 | 26 | 13 | 3 | 22 | 11 | 0 | 46 | 31 | 2 | 69 | 21 | 3 | 1 | 4 | 0 |  |
|  | acute injury prevention | 6 | 11 | 3 | 16 | 25 | 1 | 14 | 16 | 3 | 30 | 43 | 6 | 45 | 44 | 4 | 1 | 4 | 0 |  |
|  | injury rate | 8 | 12 | 0 | 18 | 24 | 0 | 18 | 13 | 2 | 29 | 45 | 5 | 61 | 31 | 1 | 3 | 2 | 0 |  |
|  | acute pain | 17 | 3 | 0 | 30 | 9 | 3 | 24 | 8 | 1 | 59 | 15 | 5 | 64 | 28 | 1 | 1 | 3 | 1 |  |
|  | chronic pain | 15 | 5 | 0 | 25 | 15 | 2 | 26 | 7 | 0 | 43 | 34 | 2 | 69 | 23 | 1 | 2 | 3 | 0 |  |
|  | blood flow | 19 | 1 | 0 | 38 | 4 | 0 | 26 | 7 | 0 | 67 | 11 | 1 | 83 | 10 | 0 | 3 | 2 | 0 |  |
|  | foam rolling hardness | 6 | 11 | 3 | 19 | 21 | 2 | 18 | 11 | 4 | 23 | 49 | 7 | 40 | 39 | 14 | 4 | 1 | 0 |  |
|  | pressure intensity | 5 | 12 | 3 | 11 | 25 | 6 | 11 | 16 | 6 | 15 | 43 | 21 | 38 | 37 | 18 | 1 | 3 | 1 |  |
| Italian-speaking countries | acute ROM | 0 | 0 | 0 | 9 | 3 | 0 | 29 | 10 | 1 | 11 | 4 | 0 | 32 | 16 | 1 | 1 | 0 | 0 |  |
|  | chronic ROM | 0 | 0 | 0 | 8 | 4 | 0 | 34 | 6 | 0 | 9 | 6 | 0 | 42 | 6 | 1 | 1 | 0 | 0 |  |
|  | acute performance | 0 | 0 | 0 | 7 | 5 | 0 | 23 | 16 | 1 | 4 | 9 | 2 | 28 | 18 | 3 | 0 | 1 | 0 |  |
|  | chronic performance | 0 | 0 | 0 | 8 | 4 | 0 | 24 | 16 | 0 | 9 | 6 | 0 | 25 | 19 | 5 | 1 | 0 | 0 |  |
|  | acute muscle stiffness | 0 | 0 | 0 | 11 | 0 | 1 | 35 | 5 | 0 | 14 | 1 | 0 | 41 | 6 | 2 | 1 | 0 | 0 |  |
|  | chronic muscle stiffness | 0 | 0 | 0 | 11 | 1 | 0 | 35 | 5 | 0 | 11 | 4 | 0 | 45 | 1 | 3 | 1 | 0 | 0 |  |
|  | fascial adhesion prevention | 0 | 0 | 0 | 11 | 1 | 0 | 27 | 10 | 3 | 12 | 3 | 0 | 40 | 8 | 1 | 1 | 0 | 0 |  |
|  | acute fascial adhesion | 0 | 0 | 0 | 12 | 0 | 0 | 33 | 6 | 1 | 11 | 4 | 0 | 39 | 9 | 1 | 1 | 0 | 0 |  |
|  | chronic fascial adhesion | 0 | 0 | 0 | 11 | 1 | 0 | 29 | 11 | 0 | 9 | 5 | 1 | 36 | 12 | 1 | 1 | 0 | 0 |  |
|  | recovery | 0 | 0 | 0 | 10 | 1 | 1 | 30 | 10 | 0 | 11 | 3 | 1 | 34 | 12 | 3 | 1 | 0 | 0 |  |
|  | acute injury prevention | 0 | 0 | 0 | 8 | 4 | 0 | 22 | 17 | 1 | 10 | 4 | 1 | 30 | 16 | 3 | 1 | 0 | 0 |  |
|  | injury rate | 0 | 0 | 0 | 7 | 5 | 0 | 24 | 16 | 0 | 10 | 4 | 1 | 34 | 11 | 4 | 1 | 0 | 0 |  |
|  | acute pain | 0 | 0 | 0 | 10 | 2 | 0 | 27 | 9 | 4 | 12 | 3 | 0 | 36 | 11 | 2 | 1 | 0 | 0 |  |
|  | chronic pain | 0 | 0 | 0 | 9 | 3 | 0 | 31 | 9 | 0 | 12 | 3 | 0 | 41 | 7 | 1 | 1 | 0 | 0 |  |
|  | blood flow | 0 | 0 | 0 | 11 | 1 | 0 | 36 | 4 | 0 | 11 | 4 | 0 | 40 | 8 | 1 | 1 | 0 | 0 |  |
|  | foam rolling hardness | 0 | 0 | 0 | 6 | 4 | 2 | 24 | 12 | 4 | 5 | 7 | 3 | 27 | 10 | 12 | 0 | 1 | 0 |  |
|  | pressure intensity | 0 | 0 | 0 | 9 | 2 | 1 | 22 | 17 | 1 | 7 | 7 | 1 | 28 | 14 | 7 | 0 | 1 | 0 |  |
| Portugal/  Spain-speaking countries | acute ROM | 5 | 1 | 0 | 1 | 2 | 0 | 1 | 0 | 0 | 7 | 3 | 0 | 2 | 1 | 1 | 1 | 0 | 0 |  |
|  | chronic ROM | 4 | 2 | 0 | 0 | 3 | 0 | 1 | 0 | 0 | 4 | 6 | 0 | 4 | 0 | 0 | 1 | 0 | 0 |  |
|  | acute performance | 4 | 2 | 0 | 1 | 1 | 1 | 1 | 0 | 0 | 2 | 8 | 0 | 1 | 1 | 2 | 1 | 0 | 0 |  |
|  | chronic performance | 2 | 4 | 0 | 1 | 2 | 0 | 1 | 0 | 0 | 1 | 9 | 0 | 3 | 0 | 1 | 1 | 0 | 0 |  |
|  | acute muscle stiffness | 4 | 2 | 0 | 2 | 1 | 0 | 1 | 0 | 0 | 7 | 3 | 0 | 1 | 3 | 0 | 1 | 0 | 0 |  |
|  | chronic muscle stiffness | 4 | 2 | 0 | 0 | 3 | 0 | 1 | 0 | 0 | 3 | 7 | 0 | 2 | 1 | 1 | 1 | 0 | 0 |  |
|  | fascial adhesion prevention | 5 | 1 | 0 | 1 | 2 | 0 | 0 | 1 | 0 | 3 | 7 | 0 | 2 | 2 | 0 | 1 | 0 | 0 |  |
|  | acute fascial adhesion | 4 | 2 | 0 | 0 | 3 | 0 | 1 | 0 | 0 | 3 | 7 | 0 | 4 | 0 | 0 | 1 | 0 | 0 |  |
|  | chronic fascial adhesion | 5 | 1 | 0 | 0 | 2 | 1 | 1 | 0 | 0 | 4 | 6 | 0 | 3 | 1 | 0 | 1 | 0 | 0 |  |
|  | recovery | 4 | 2 | 0 | 1 | 2 | 0 | 1 | 0 | 0 | 4 | 6 | 0 | 2 | 2 | 0 | 1 | 0 | 0 |  |
|  | acute injury prevention | 2 | 4 | 0 | 1 | 2 | 0 | 1 | 0 | 0 | 2 | 7 | 1 | 1 | 2 | 1 | 1 | 0 | 0 |  |
|  | injury rate | 3 | 3 | 0 | 1 | 2 | 0 | 1 | 0 | 0 | 3 | 7 | 0 | 3 | 0 | 1 | 1 | 0 | 0 |  |
|  | acute pain | 4 | 2 | 0 | 3 | 0 | 0 | 1 | 0 | 0 | 9 | 1 | 0 | 2 | 1 | 1 | 1 | 0 | 0 |  |
|  | chronic pain | 3 | 3 | 0 | 2 | 1 | 0 | 1 | 0 | 0 | 4 | 6 | 0 | 2 | 1 | 1 | 1 | 0 | 0 |  |
|  | blood flow | 6 | 0 | 0 | 1 | 1 | 1 | 1 | 0 | 0 | 8 | 1 | 1 | 4 | 0 | 0 | 1 | 0 | 0 |  |
|  | foam rolling hardness | 2 | 4 | 0 | 2 | 1 | 0 | 1 | 0 | 0 | 3 | 6 | 1 | 2 | 1 | 1 | 1 | 0 | 0 |  |
|  | pressure intensity | 3 | 3 | 0 | 1 | 1 | 1 | 1 | 0 | 0 | 1 | 7 | 2 | 3 | 0 | 1 | 1 | 0 | 0 |  |
| English-speaking countries | acute ROM | 0 | 2 | 0 | 6 | 0 | 0 | 2 | 0 | 0 | 23 | 3 | 0 | 1 | 1 | 0 | 0 | 0 | 0 |  |
|  | chronic ROM | 0 | 2 | 0 | 6 | 0 | 0 | 0 | 2 | 0 | 11 | 15 | 0 | 2 | 0 | 0 | 0 | 0 | 0 |  |
|  | acute performance | 1 | 0 | 1 | 2 | 4 | 0 | 1 | 1 | 0 | 9 | 13 | 4 | 0 | 2 | 0 | 0 | 0 | 0 |  |
|  | chronic performance | 1 | 1 | 0 | 1 | 5 | 0 | 0 | 2 | 0 | 6 | 20 | 0 | 1 | 1 | 0 | 0 | 0 | 0 |  |
|  | acute muscle stiffness | 2 | 0 | 0 | 4 | 2 | 0 | 2 | 0 | 0 | 22 | 4 | 0 | 2 | 0 | 0 | 0 | 0 | 0 |  |
|  | chronic muscle stiffness | 0 | 2 | 0 | 1 | 5 | 0 | 0 | 2 | 0 | 12 | 14 | 0 | 2 | 0 | 0 | 0 | 0 | 0 |  |
|  | fascial adhesion prevention | 1 | 1 | 0 | 1 | 5 | 0 | 1 | 1 | 0 | 14 | 12 | 0 | 1 | 1 | 0 | 0 | 0 | 0 |  |
|  | acute fascial adhesion | 0 | 2 | 0 | 2 | 4 | 0 | 0 | 2 | 0 | 13 | 13 | 0 | 2 | 0 | 0 | 0 | 0 | 0 |  |
|  | chronic fascial adhesion | 0 | 2 | 0 | 0 | 6 | 0 | 0 | 2 | 0 | 11 | 15 | 0 | 1 | 1 | 0 | 0 | 0 | 0 |  |
|  | recovery | 2 | 0 | 0 | 2 | 4 | 0 | 2 | 0 | 0 | 13 | 11 | 2 | 2 | 0 | 0 | 0 | 0 | 0 |  |
|  | acute injury prevention | 0 | 2 | 0 | 0 | 6 | 0 | 2 | 0 | 0 | 9 | 17 | 0 | 1 | 1 | 0 | 0 | 0 | 0 |  |
|  | injury rate | 0 | 2 | 0 | 0 | 6 | 0 | 0 | 2 | 0 | 10 | 16 | 0 | 1 | 1 | 0 | 0 | 0 | 0 |  |
|  | acute pain | 2 | 0 | 0 | 4 | 2 | 0 | 2 | 0 | 0 | 19 | 6 | 1 | 1 | 1 | 0 | 0 | 0 | 0 |  |
|  | chronic pain | 1 | 1 | 0 | 3 | 3 | 0 | 0 | 2 | 0 | 13 | 13 | 0 | 1 | 1 | 0 | 0 | 0 | 0 |  |
|  | blood flow | 1 | 1 | 0 | 4 | 2 | 0 | 2 | 0 | 0 | 22 | 4 | 0 | 1 | 1 | 0 | 0 | 0 | 0 |  |
|  | foam rolling hardness | 1 | 1 | 0 | 2 | 4 | 0 | 1 | 1 | 0 | 15 | 11 | 0 | 1 | 1 | 0 | 0 | 0 | 0 |  |
|  | pressure intensity | 1 | 0 | 1 | 0 | 6 | 0 | 2 | 0 | 0 | 12 | 11 | 3 | 1 | 1 | 0 | 0 | 0 | 0 |  |

| Country | Topic | Therapy  setting | | | Sport  science | | | Coach/  Trainer | | | Multiple | | | No practical  experience | | | Other | | |  |
| --- | --- | --- | --- | --- | --- | --- | --- | --- | --- | --- | --- | --- | --- | --- | --- | --- | --- | --- | --- | --- |
|  |  |  |  |  |  |  |  |  |  |  |  |  |  |  |  |  |  |  |  |  |
|  |  | + | - | o | + | - | o | + | - | o | + | - | o | + | - | o | + | - | o |  |
| German-speaking  countries | Contraindication | 19 | 1 | 0 | 29 | 13 | 0 | 21 | 12 | 0 | 56 | 23 | 0 | 52 | 41 | 0 | 1 | 4 | 0 |  |
|  | Recommendation | 9 | 5 | 6 | 13 | 11 | 18 | 18 | 6 | 9 | 27 | 18 | 34 | 36 | 19 | 38 | 1 | 1 | 3 |  |
| Italian-speaking  countries | Contraindication | 0 | 0 | 0 | 5 | 7 | 0 | 18 | 22 | 0 | 7 | 8 | 0 | 15 | 34 | 0 | 0 | 1 | 0 |  |
|  | Recommendation | 0 | 0 | 0 | 7 | 2 | 3 | 31 | 1 | 8 | 10 | 1 | 4 | 35 | 1 | 13 | 0 | 0 | 1 |  |
| Portugal/  Spain-speaking  countries | Contraindication | 4 | 2 | 0 | 1 | 2 | 0 | 0 | 1 | 0 | 5 | 5 | 0 | 3 | 1 | 0 | 1 | 0 | 0 |  |
|  | Recommendation | 4 | 2 | 0 | 1 | 2 | 0 | 1 | 0 | 0 | 3 | 3 | 4 | 2 | 1 | 1 | 0 | 0 | 1 |  |
| English-speaking  countries | Contraindication | 2 | 0 | 0 | 3 | 3 | 0 | 1 | 1 | 0 | 11 | 15 | 0 | 0 | 2 | 0 | 0 | 0 | 0 |  |
|  | Recommendation | 1 | 1 | 0 | 2 | 3 | 1 | 1 | 0 | 1 | 16 | 6 | 4 | 1 | 0 | 1 | 0 | 0 | 0 |  |

Table 3S Distribution (relative distribution within a profession) of responses by question and profession

| **Topic** | **Sport science** | | | **Practical background** | | | **Other** | | | |
| --- | --- | --- | --- | --- | --- | --- | --- | --- | --- | --- |
|  | **positive**  **effect** | **no effect** | **negative**  **effect** | **positive**  **effect** | **no effect** | **negative**  **effect** | **positive**  **effect** | **no effect** | **negative**  **effect** |  |
| **fascial adhesion**  **prevention ^a,b^** | 39  (61.9) | 23  (36.5) | 1  (1.6) | 155  (66.2) | 75  (32.1) | 4  (1.7) | 123  (79.4) | 31  (20.0) | 1  (0.7) |  |
| **chronic fascial adhesion ^a,b^** | 36  (57.1) | 26  (41.3) | 1  (1.6) | 139  (59.4) | 93  (39.7) | 2  (0.9) | 123  (79.4) | 30  (19.4) | 2  (1.3) |  |
| **acute injury prevention ^a^** | 25  (39.7) | 37  (58.7) | 1  (1.6) | 98  (41.9) | 121  (51.7) | 15  (6.4) | 80  (51.6) | 67  (43.2) | 8  (5.2) |  |
| **acute injury rate ^a,b^** | 26  (41.3) | 37  (58.7) | 0  (0.0) | 106  (45.3) | 120  (51.3) | 8  (3.4) | 104  (67.1) | 45  (29.0) | 6  (3.9) |  |

Abbreviation: ROM = range of motion,

^a^ χ² goodness of fit test, significant difference (p<0.05) between distributions
^b^ χ² test of independence, significant difference (p<0.05) between professions

Table 4S Distribution of responses by question and countries

| **Topic** | **German-speaking countries** | | | **Italian-speaking countries** | | | **Portuguese-/Spanish-speaking countries** | | | **English-speaking countries** | | | |
| --- | --- | --- | --- | --- | --- | --- | --- | --- | --- | --- | --- | --- | --- |
|  | **positive effect** | **no effect** | **negative effect** | **positive effect** | **no effect** | **negative effect** | **positive effect** | **no effect** | **negative effect** | **positive effect** | **no effect** | **negative effect** |  |
| **fascial adhesion prevention ^a,b^** | 196  (72.1) | 74  (27.2) | 2  (0.7) | 91  (77.8) | 22  (18.8) | 4  (3.4) | 12  (48.0) | 13  (52.0) | 0  (0.0) | 18  (47.4) | 20  (52.6) | 0  (0.0) |  |
| **chronic fascial adhesion ^a,b^** | 186  (68.4) | 84  (30.9) | 2  (0.7) | 86  (73.5) | 29  (24.8) | 2  (1.7) | 14  (56.0) | 10  (40.0) | 1  (4.0) | 12  (31.6) | 26  (68.4) | 0  (0.0) |  |
| **acute injury prevention ^a,b^** | 112  (41.2) | 143  (52.6) | 17  (6.3) | 71  (60.7) | 41  (35.0) | 5  (4.3) | 8  (32.0) | 15  (60.0) | 2  (8.0) | 12  (31.6) | 26  (68.4) | 0  (0.0) |  |
| **acute injury rate ^a,b^** | 137  (50.4) | 127  (46.7) | 8  (2.9) | 76  (65.0) | 36  (30.8) | 5  (4.3) | 12  (48.0) | 12  (48.0) | 1  (4.0) | 11  (28.9) | 27  (71.1) | 0  (0.0) |  |

Abbreviation: ROM = range of motion

^a^ χ² goodness of fit test, significant difference (p<0.05) between distributions
^b^ χ² test of independence, significant difference (p<0.05) between countries
